# Coordination Dynamics and Thermal Stability with Aminal Metallogels and Liquids

**DOI:** 10.3390/polym11081237

**Published:** 2019-07-26

**Authors:** Peter J. Boul, Diana K. Rasner, Peter D. Jarowski, Carl J. Thaemlitz

**Affiliations:** Aramco Services Company, Aramco Research Center, 16300 Park Row Blvd. Houston, Houston, TX 77084, USA

**Keywords:** constitutional dynamics, dynamers, high temperature

## Abstract

In this article, we review a dynamic covalent gel system developed as a high temperature well construction fluid. The key gel/fluid phase changes and related materials properties are addressable via the constitutional and coordination dynamics of the equilibrium and non-equilibrium molecular species comprising the material. The interplay between these species and external stimuli leads to material adaptability. Specifically, the introduction of metal ions into a non-equilibrium hemiaminal gel reverts this phase into a non-equilibrium liquid. When heated, this liquid transforms itself catalytically into the thermodynamically favoured closed-ring polyhexahydrotriazine (PHT) gel product. The temperature stability of different PHT gel formulations is evaluated as a function of the inclusion of various salts. It is possible to revert this thermodynamic PHT gel back into a liquid. This pH dependent transformation depends on the R groups linking the hexahydrotriazines (HTs) to one another. While polyethylene glycol (PEG) based PHT gels revert to liquids with water and mild protonation conditions, in comparison, polypropylene glycol (PPG) based gels require stronger acid conditions with heat, or a different more nucleophilically driven ring-opening mechanism by, for example, phosphines. The covalent dynamic chemistry in this chemical system gives way to many possible applications in addition to the high temperature solution-gelation (sol-gels) for which it has been primarily designed.

## 1. Introduction

Dynamic functionality in materials design can bring about adaptability and programmed responsivity when imparted through inherently stimulus-responsive constitutionally dynamic building blocks [1,2,3]. Because smart chemical systems engineered with constitutionally dynamic materials (CDMs) can be made to change their physical properties remotely and through real-time instruction, they hold substantial promise across many industries. For example, in aerospace, sensing and sealing fractures remotely has significant benefits in structures that are difficult to access for repair [4]. Controlled and triggered release chemistries find applications in medicine [5], cosmetics [6], food, agriculture [7], and energy. In the oil and gas industry, wells can require the deployment of high performance materials to depths exceeding 12,000 m [8]. These depths come with substantial materials challenges regarding pressure and temperature tolerances. Furthermore, the remoteness of oil and gas reservoirs and well construction materials brings certain value to dynamic chemistries, especially for their inherent capacity to be controlled remotely.

We found that polyhexahydrotriazines (PHTs) offer the possibility to broaden the thermal performance window of oil and gas well construction fluids where the remote control of material phase transformations with high temperature stability is a requirement [9]. The potential of PHTs in the industry, however, is not limited to oil well construction technologies. We have previously demonstrated the possibility of using these materials for controlled triggered additive release [10], reversible gelling fluids, and self-healing polymer gels. In this article, we describe the dynamic chemistry of PHTs to highlight the effect of metals on the thermodynamics and kinetics of the condensations between amines and aldehydes to hemiaminal gels, and finally the PHT gel phase [11].

Aminal functionalities, such as those in hexahydrotriazines (HTs), are of value, in part, for their ability to undergo functional group transformations in response to pH changes and metal coordination [12]. When these functionalities are brought into polymer networks, the functional group transformations correspond to network rearrangements, in some cases leading to solution-gelation (sol-gel) dynamics regulated by the aminal chemistry. Condensation products of polyalkoxy multiamines or dianiline compounds and formaldehyde to hemaminal intermediates and to polyHTs have been shown to be fully reversible with pH and therefore make for attractive recyclable gels and thermosets [13,14]. The thermodynamics and kinetics of these CDMs is highlighted in this paper, bringing experimental results in correspondence with density functional theory (DFT) computational analysis. In addition, these phase changes are examined under simulated well conditions of high pressure and temperature. The thermal stability of the final PHT is very much dependent on certain catalytic and stabilizing salts introduced to the gel formulations. These will be discussed in detail along with a review of the role of metals in catalysing the hexahydrotriazine (HT) formation from hemiaminal precursor products.

## 2. Background and Mechanisms for HT Dynamism

**Scheme 1:**
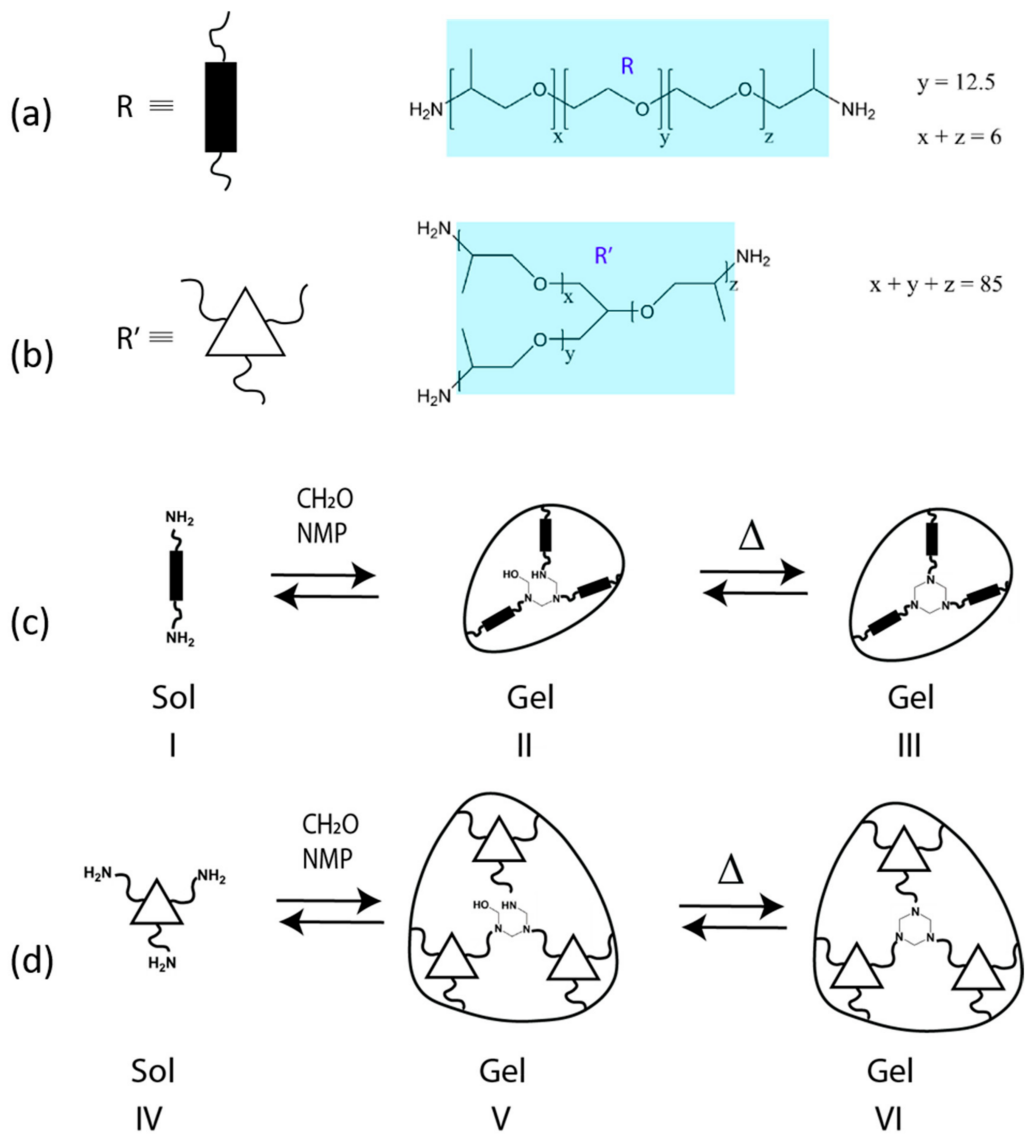
(**a**) The 900 Da polypropylene glycol capped polyethylene glycol diamine, Jeffamine^®^ ED-900. (**b**) The 5000 Da polypropylene glycol triamine, Jeffamine^®^ T-5000. (**c**) The reaction of Jeffamine ED-900 with formaldehyde in *N*-methyl pyrrolidone (NMP). (**d**) The reaction of Jeffamine T-5000 with formaldehyde in NMP. Sol, solution; gel, gelation.

Scheme 1a shows the structure of Jeffamine^®^ ED900, which is a 900 Dalton (Da), polypropylene glycol capped polyethylene glycol diamine. Scheme 1b shows the structure of Jeffamine^®^ T-5000, which is a 5000 Da, polypropylene glycol triamine. Scheme 1 illustrates the molecular structure of the gels in the absence of metals as they are formed from the condensation of formaldehyde and the two different amine-terminated polymers, illustrated in Scheme 1a,b. The initial gel forming reaction occurs upon mixing the cracked paraformaldehyde in a polar aprotic solvent, such as *N*-methyl pyrrolidone (NMP) [14]. The reactions leading to products II and V occur readily and at room temperature. This acyclic hemiaminal is believed to be responsible for the initial gel state observed at room temperature. The ring-closing reaction leading to products III and VI requires either heating under pressure (i.e., ≥3 MPa) or mild heating (i.e., ≥70 °C) in the presence of a catalyst [11].

**Scheme 2:**
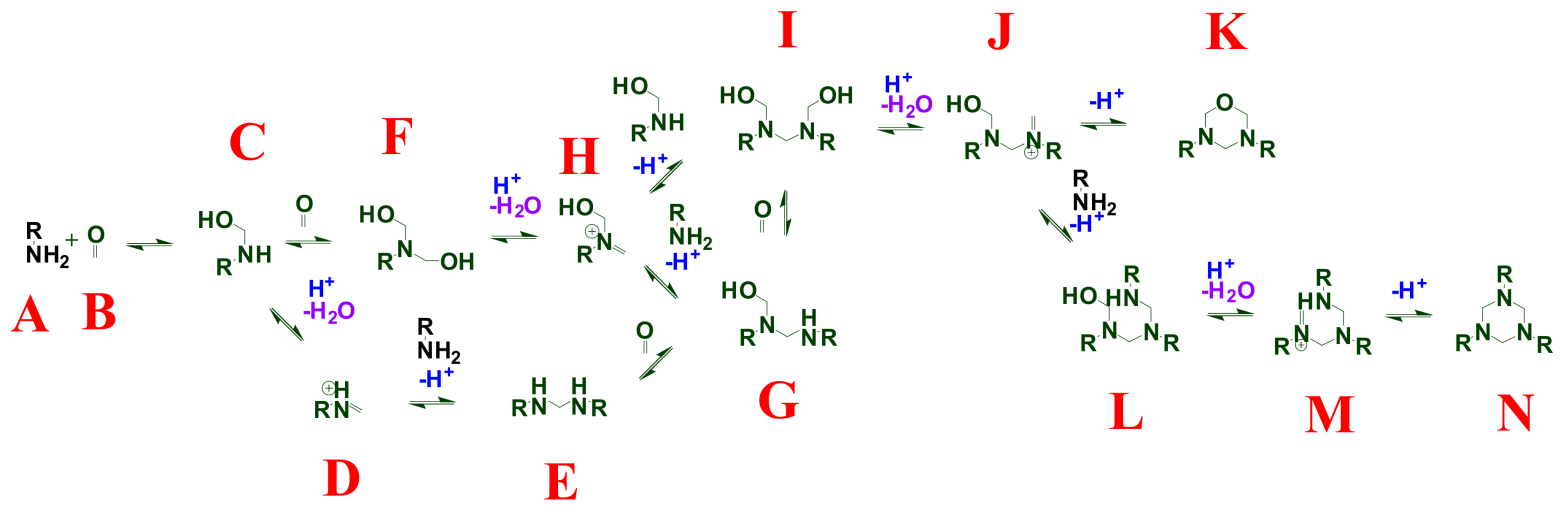
Mechanism for the condensation of formaldehyde with organic amines producing a series of aminals and hemiaminals. The solvents that have been tested for this chemistry are NMP, *N*,*N*’-dimethylformamide (DMF), and dimethylsulfoxide (DMSO) [11].

**Scheme 3:**
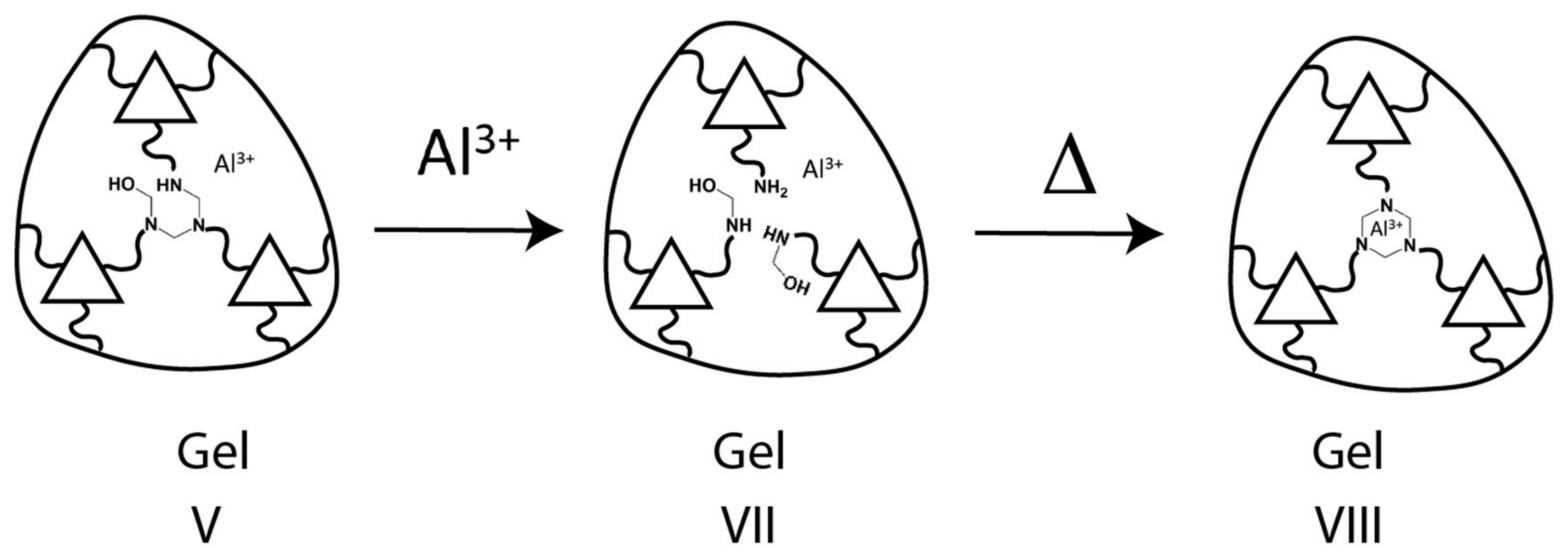
From the dynamics of the gel break with aluminum along with heating, we proposed the mechanism for gel breaking and reformation to be as illustrated here.

More details of the reaction pathway of the amine-aminal condensation described in Scheme 1c,d are provided in Scheme 2. Jones and co-workers determined that the condensation of these materials at room temperature would yield to product L in Scheme 2 (products II and V in Scheme 1). There is then a kinetic barrier of 27 kcal/mol to reach product N (products III and VI in Scheme 1), which, at 3 kcal/mol lower in energy than product L, is the lowest free energy product in Scheme 2 [15]. We have found that the introduction of trivalent metal salts (such as aluminium chloride) to CDMs of polyalkoxy multiamines and formaldehyde in polar aprotic solvent shows a kinetically governed reversion of hemiaminal networked polymer gels from gel to sol. Our proposed mechanism for this transformation is presented in Scheme 3. As from Scheme 3, the conversion of the hemiaminal gel (V) to a liquid (VII) can be followed by a transformation to gel (VIII) through mild heating (i.e., 70 °C for a few hours). This reaction can be reversible with a pH change. This thermodynamically favoured product (VIII) can also be reverted from gel to liquid through nucleophilic attack by a thiol- [16] or phosphine-based [10] reagent.

**Scheme 4:**
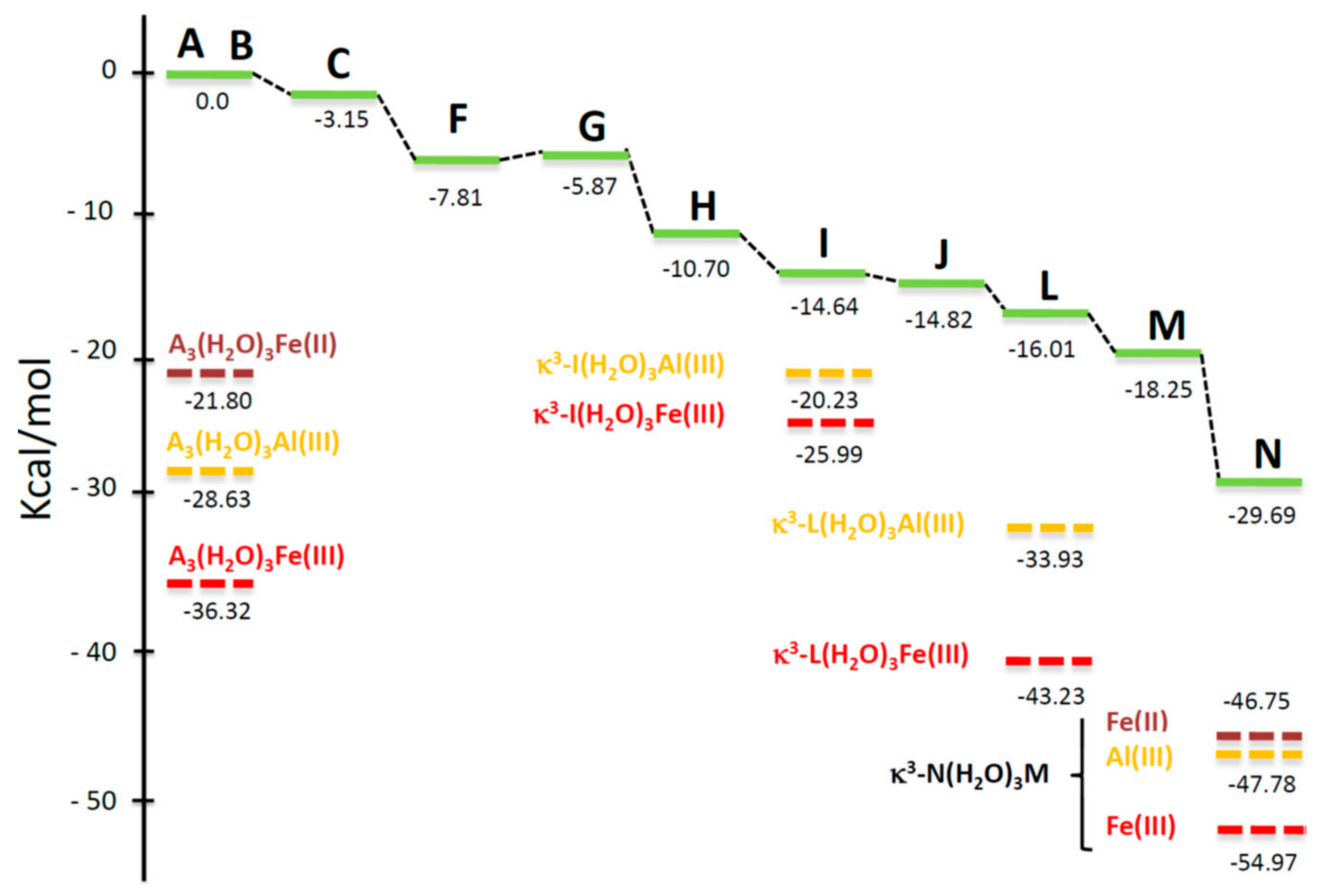
M06-2x/6-31+g(d)//B3LYP/6-31+g(d) (COSMO in water) reaction free-energy coordinate (ΔG, kcal/mol) for the different intermediates drawn in Scheme 2. Reprinted (adapted) with permission from [11]. Copyright 2017 American Chemical Society.

From our earlier work on the topic, comparing the relative energies of the complexes with aluminium (Scheme 4) reveals a shift in the energetic landscape as this constitutionally dynamic system adapts to the presence of the metal cation. Product N remains the lowest energy product and in fact appears to be further stabilized by association with the metal. This is the product that is achieved over time or with mild heating (to 70 °C, for instance). Pictures of the different liquids and gels described in Scheme 3 are provided in the supporting information, Figure S1. The addition of more metal was found to slow the reaction as the metal stabilizes the starting materials and earlier, less networked reaction intermediates. The relative molar equivalents of aluminum chloride to the triamine increased from 1.1 equivalents to 2.3 equivalents giving sol-gel transitions in the range of 0.6 to 6.3 h, correspondingly [11].

Insights into the role of aluminum chloride for catalysis (through computational analysis) along with supporting experimental information depicting relative reaction rate enhancement are presented through the experiments described in detail in this paper. Further examination of the high temperature and high-pressure stability of this gel system is also presented. We have also uncovered gel compositions that allow for higher temperature tolerance than previously observed, through the inclusion of various monovalent salts. The results of these initial investigations, widening the performance window of this product driven by upstream oil and gas applications, are presented.

## 3. Materials and Methods

### 3.1. Instrument Descriptions

#### 3.1.1. Pressurized Rheological Instrumentation

Pressurized shear modulus rheological measurements were performed on a Grace M5600 HPHT rheometer (Grace Instrument, Houston, TX, USA) with a circulating oil bath heater and pressurized with nitrogen to 3.4 MPa. 

Pressurized rotational rheology measurements were performed using a Fann iX77 HPHT rheometer (Fann Instrument Company, Houston, TX, USA) with built in electric heater and pressurized using hydraulic fluid.

#### 3.1.2. Nuclear Magnetic Resonance (NMR) Instrumentation

NMR spectroscopy was performed using a Bruker Aeon™ 500 spectrometer utilizing TopSpin™ 3.2 (Bruker BioSpin, Billerica, MA, USA). ^1^H-NMR spectra were collected at 500 MHz. All spectra were obtained from 1–5% (w/v) 0.7 mL solutions in d_7_-*N*,*N*’-dimethylformamide (DMF).

### 3.2. Chemicals

NMP, paraformaldehyde, and aluminum chloride hexahydrate were purchased from Fisher Scientific Co. LLC (Thermo Fisher Scientific, Waltham, MA, USA). Jeffamine^®^ T-5000 and Jeffamine^®^ ED-900 were obtained from Huntsman International LLC (Huntsman Corporation, The Woodlands, TX, USA). Deuterated d_7_-DMF was obtained from Acros Organics (Thermo Fisher Scientific). Calcium bromide hydrate, calcium chloride dihydrate, calcium chloride hexahydrate, cesium chloride, sodium bromide, and zinc bromide were all obtained from Sigma Aldrich (Milipore Sigma, St. Louis, MO, USA).

### 3.3. Methods

#### 3.3.1. Hemiaminal (Kinetic) Gel Preparation Procedure

NMP and paraformaldehyde (in the proportions described in Table 1) were stirred together at 70 °C in an oil bath on a heated stir plate for 30 min. A polyoxypropylene triamine (Jeffamine^®^ T-5000 from Huntsman Corporation) was then added to the mixture and allowed to continue to stir at 70 °C for another 30 min. The mixture was then removed from the oil bath and allowed to cool to room temperature where a gel would form, referred to in this paper as ‘65—5 kinetic gel’ (from the ratio of NMP to paraformaldehyde, where the ratio of paraformaldehyde to Jeffamine^®^ T-5000 was 5.4:1). 

#### 3.3.2. Transformation of the Hemiaminal (kinetic) Gel to Liquid Phase—Method for the Transformation of VII to VIII in Scheme 3

The gel from above was cut into smaller pieces and placed into a jar with a magnetic stir rod. Aluminium chloride hexahydrate in the amount indicated in Table 1 was added into the jar with the pieces of gel and allowed to stir for 24 h, where breakdown of the gel into a liquid would occur. 

#### 3.3.3. Transformation of the Liquid Phase to the PHT (thermodynamic) Gel—Method for the Transformation of VIII to IX in Scheme 3

After the gel was reverted to a liquid, it was then heated to 70 °C, where the second gelation time of the material (reaction c in Scheme 1) was observed. These transitions were examined rheologically with temperature and pressure.

#### 3.3.4. Pressurized Rheological Study under Elevated Temperature—Simulation of the Well Environment

After the addition of aluminium to the hemiaminal gels and following the transformation of the materials into liquids, the gel times of the materials were observed in a Grace M5600 HPHT rheometer at 70, 100, and 150 °C at 3.4 MPa. The rheometer uses an oscillatory test where the sample was subjected to a steady 1 Hz frequency and 100% amplitude oscillatory strain and the resulting stress was measured and recorded as an elastic and viscous modulus (G’ and G”, respectively). Gel time was interpreted as when G’ crosses over G”.

#### 3.3.5. Gel Preparation and Gel Breaking for Polyethylene Glycol (PEG) Multiamine versus Polypropylene Glycol (PPG) Multiamine Reactivity Comparison

NMP and paraformaldehyde (in the proportions described in Table 2) were stirred together at 70 °C in an oil bath on a heated stir plate for 30 min. A polyoxyethylene diamine (Jeffamine^®^ ED900 from Huntsman Corporation) was then added to the mixture and allowed to continue to stir at 70 °C for another 30 min. The mixture was then removed from the oil bath and allowed to cool to room temperature, where a gel would form. Addition of aluminium chloride hexahydrate rendered the liquid phase as found in 2.3.2, from which additional heating would yield the equivalent PHT.

Reversibility of the formation of PHT gels (reversion to the sol state) made with PPG (2.3.3) versus PEG (2.3.4) was studied under multiple conditions (Table 3). To a freshly made PHT gel broken up into smaller pieces, as described in 2.3.2, 1.0 g of tris(2-carboxyethyl)phosphine (TCEP) is added and allowed to ‘break’ into a liquid overnight. For phosphine and NMP as a breaker, 1.0 g of TCEP was dissolved in 10 mL of NMP and added to a freshly made PHT gel. Strong acid and heat was tested as a breaker by adding 10mL of concentrated HCl to a PHT gel and allowed to sit in an oil bath at 70 °C overnight. Further, 10 mL of DI water added to a PHT gel was also tested as a breaker.

#### 3.3.6. NMR Model Compound Preparation

To probe into greater detail the mechanism for the amine-aminal dynamics of the system, we analyze the reactivity with NMR, replacing the polymeric amines used in the gel system with a short-chain alkyl-amine. The choice of this alkyl-amine over Jeffamine^®^ was made because the use of Jeffamine^®^ broadens the NMR spectra, obscuring valuable information about the aminal dynamics. The method for these studies follows. 

In a vial with a magnetic stir bar, 1 g of d_7_-DMF was stirred with 0.0312 g of paraformaldehyde at 70 °C in an oil bath for 30 min. The vial was then removed from the heat to cool to room temperature. In a separate vial, 0.0420 g of butylamine was weighed out and set aside. An additional 0.0557 g of aluminum chloride hexahydrate was added to the butylamine vial for the aluminum kinetics study at this step.

A bath of dry ice and acetonitrile (−41 °C) was made and the two vials along with a clean empty NMR tube were placed into the bath to cool for 10 min. Using a syringe and needle, the DMF/paraformaldehyde mixture was quickly transferred and mixed into the cold butylamine vial. This mixture was then quickly transferred via syringe and needle into the cold clean NMR tube and allowed to remain there until ready to measure. With the NMR sample chamber stabilized at −25 °C, the NMR sample was quickly removed from the dry ice bath, the sides wiped down clean, and the sample was placed into the NMR for kinetic study. Once sufficient data were collected at −25 °C, the NMR temperature was raised to 0 °C, re-shimmed, and data collection continued.

In the NMR analysis, the integral area of the aldehydic DMF-d7 (solvent) proton at 8.03 ppm was set to 1.0 for all spectra. All integral areas were relative to this signal. This helps to distinguish changes in concentrations of molecular species as they form or disappear with time.

#### 3.3.7. High Temperature Stability Gels Procedure

NMP and the following salts were stirred with a magnetic stir bar until full saturation at room temperature occurs. The densities of NMP salt solutions were as follows in Table 4.

To a vial with a stir bar, 41.2 g of the saturated NMP salt solutions above was stirred with 1.04 g of paraformaldehyde at 70 °C for 30 min. A total of 32.0 g of Jeffamine T5000 was added to the mixture and allowed to stir for an additional 3 h, where a stable thermodynamic PHT gel would form.

With regards to this observational experiment, stability is defined as the presence of liquid seen when holding the gel system (contained in a glass vial) upside down. Absence of liquid was considered stable. The PHT gel systems once formed and checked for stability at 70 °C were immediately placed into an oven preset at 150 °C to test for stability at high temperature conditions over time.

#### 3.3.8. Computational Methods

Density functional theory computations were carried out using the software NWChem. All molecular structures were optimized using the Becke-Li-Yang Parr hybrid functional (B3LYP) with vibrational frequency analysis and thermal corrections calculated on these structures in the gas-phase. For HCNO atoms, the split-valence basis set with diffuse functions (6-31+G(d)) was applied, whereas for the aluminum and iron centers, an effective core potential (ECP) with the LANL2DZ basis set was used. Single-point calculations using the meta generalized gradient approximation M06-2x functional were applied with the COSMO continuum solvation model for water (dielectric constant of 78). Water was chosen as the solvent as it is a significant by-product of the hemi-aminal mechanism and should be present in large amounts in the reaction mixture. Small amounts of water will drive the solvent polarity significantly. The final level of theory is M06-2x/6-31+G(d) with COSMO solvent (water)//B3LYP/6-31+G(d) for HCNO and M06-2x/LANL2DZ with COSMO solvent//B3LYP/LANL2DZ for metal centers. All energies presented in the main text are free-energies in solvo at 298.15 K at this level on the B3LYP/6-31+G(d) optimized structures with corresponding harmonic frequencies taken from the gas-phase. Optimization of the global minimum energy structures for all organic ligands involved in the hemi-aminal mechanism with R = Methyl was done using the ChemAlive (www.chemalive.com) automation code, Consru*Qt*, to treat the conformational and tautomeric space of the intermediate structures.

## 4. Results and Discussion

### 4.1. Overview of Previously Reported Gel Dynamics

**Figure 1:**
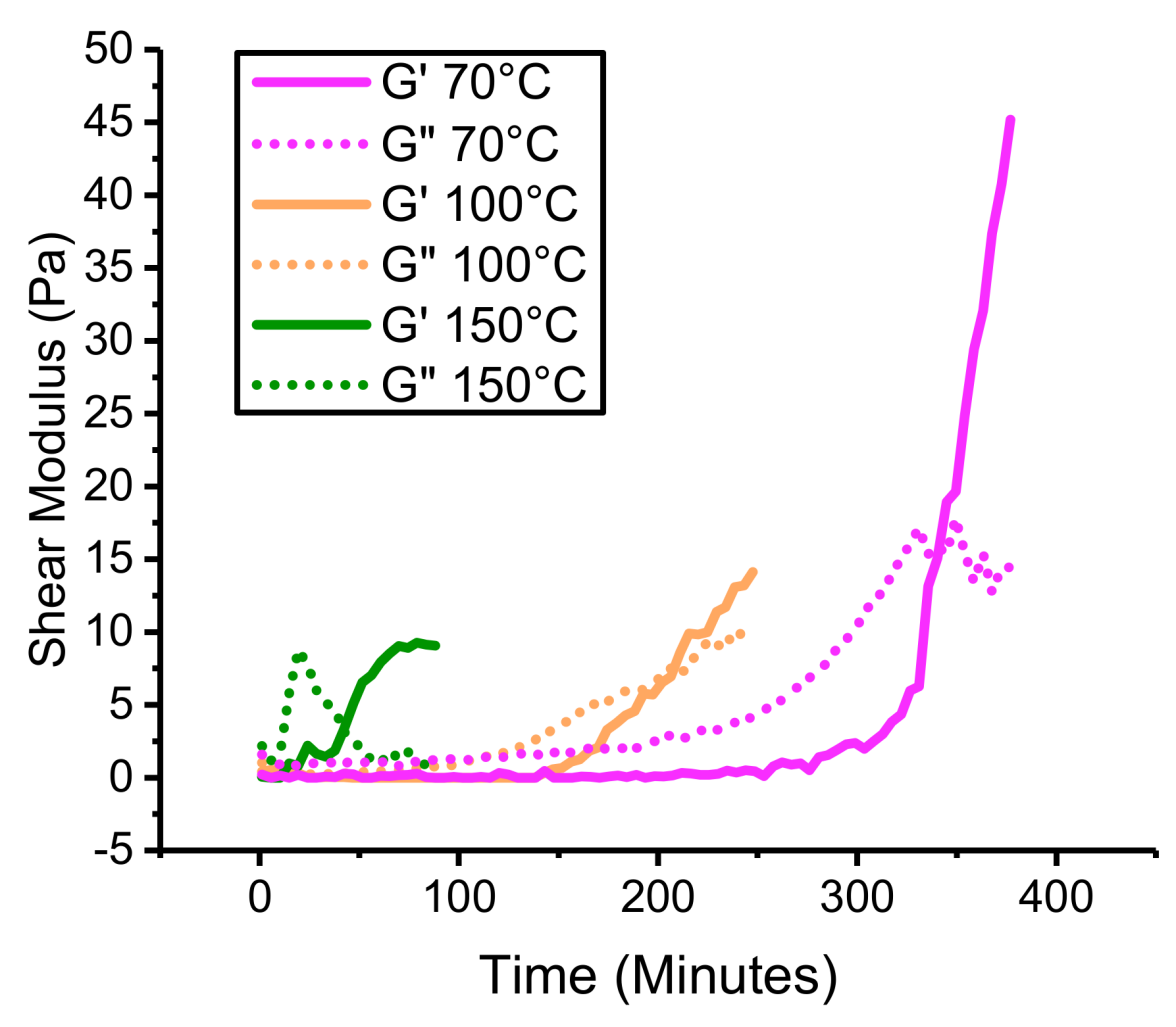
The 65—5 liquid (formulation described in Table 1) is heated to three different temperatures (70, 100, and 150 °C) at 3.4 MPa, simulating three different wellbore environments. The data clearly show a decrease in gel time with increasing temperature and the thermal tolerance of the gel transitions out to 150 °C. Reprinted (adapted) with permission from [9]. Copyright (2018) American Chemical Society.

To test if these sol-gel transitions can be utilized in the oil field, these functional dynamics were tested at temperatures up to 150 °C and pressures up to 60 MPa. As expected, a rise in temperature leads to a faster rate of gelation for the PHT system, as described in Table 1. Three 65—5 liquid samples were tested in a Grace M5600, where pressure was maintained at 3.4 MPa and the system was heated to varying temperatures. The data presented in Figure 1 are from small amplitude oscillatory shear experiments, where G’ is the elastic modulus of the materials tested and G” is the viscous modulus. The cross-over points of G’ and G” depict the sol-gel transition. For this 65—5 gel, we see the sol-gel transition times decrease with increasing temperature. At 70, 100, and 150 °C, the sol-gel transitions occur at 350, 200, and 50 min, respectively. The transformation occurring at temperatures up to 150 °C indicates, in part, the suitability for this chemical system under higher temperature wellbore situations.

**Figure 2:**
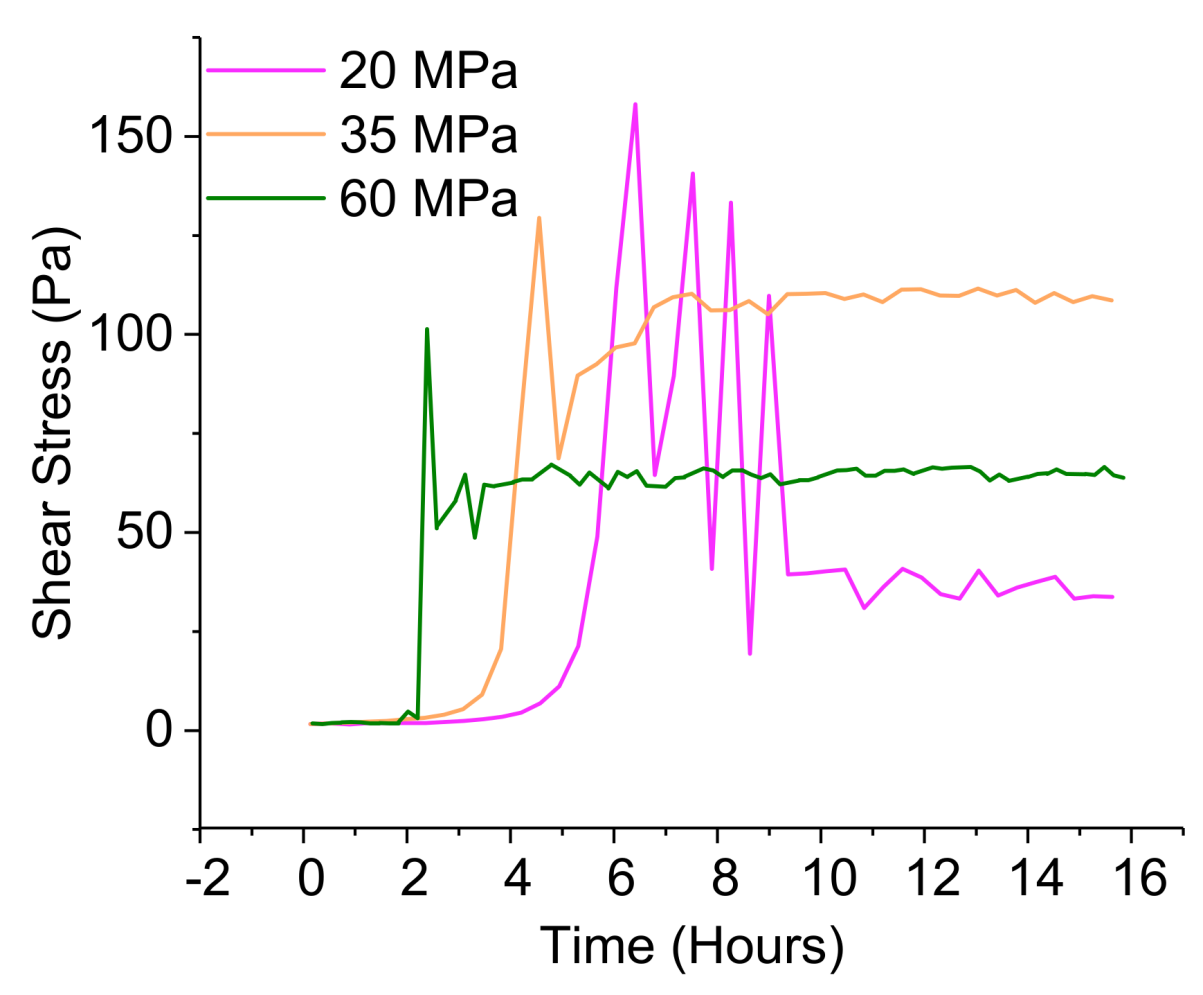
The 65—5 liquid (formulation described in Table 1) is heated to 70 °C and pressurized to 20, 35, and 60 MPa. The data clearly show a decrease in gel time with increasing pressure, suitable to more extreme wellbore environments. Reprinted (adapted) with permission from [9]. Copyright (2018) American Chemical Society.

The 65—5 system was also tested for its sol-gel transition under much higher pressures with rotational rheology, further simulating some of the more extreme conditions found in the oil field. While holding the temperature steady at 70 °C, the 65—5 liquid is pressurized to 20, 35, and 60 MPa and monitored for when the sol-gel transition occurs under rotational shear, as seen in Figure 2. When the pressure of system is increased while holding temperature constant, the sol-gel transition time of the 65—5 system decreases to 390, 270, and 140 min, respectively. This indicates that pressure has a direct effect on the kinetics of the 65—5 sol-gel transition and accelerates the system towards the condensation pathway of increasingly branched hemiaminal and aminal condensation products. The unique property of the PHT system to make these sol-gel transitions at a wide range of temperatures and pressures shows its versatility for use in oil well completion projects.

### 4.2. PHT Breakdown with Water

A distinct advantage to using this constitutionally dynamic gel system lies in its reversibility and the ability to remove said gel from wherever it is being placed via a “triggered release”. We previously reported that a PHT gel formed with our original 65—5 formula could “break” under elevated temperatures and pressures using phosphine. However, a more environmentally friendly and easier to use agent for breaking can be achieved by replacing the high molecular weight PPG triamine in the original 65—5 formulation for a smaller weight PEG diamine. 

The reversibility of PEG amine PHTs, as compared with PPG amine PHTs, is notably similar in these condensation polymers with the large exception of water being used as a breaker. This is largely because of the high solubility of the 900 Da PEG diamine and the poor solubility of 5 kDa PPG triamine in water. When water is added to an PHT gel of PEG diamine, the system reverts to liquid within minutes. On the other hand, the PPG triamine remains unchanged in the presence of the same quantity of water. The most effective way to revert the PHT gel of the PPG triamine was found to be through the use of a saturated solution (10% wt) of phosphine in NMP, in this case (tris(2-carboxyethyl)phosphine).

### 4.3. Thermal Stability of PHT

We have previously reported the addition of trivalent metal salts (FeCl_3_, AlCl_3_) gives us the lowest energy and most stabilized products in our PHT scheme, either overtime or in the presence of heat. This led us to evaluate what other metal salts could further stabilize this scheme and to what extent. To evaluate the limits of the thermal stabilities of the HT gels, we assayed a variety of different salts to observe how the salt systems may influence the gel stabilities. A summary of gel thermal stabilities is presented in Table 5.

The salts tested are often used in completion brines for such purposes as density adjustment. Interestingly, the various salts influence thermal stability of the gels differently. From our investigations of aluminium chloride, calcium halides, zinc bromide, cesium salts, and sodium bromide, we found that the greatest stability was observed in the case of sodium bromide. In all cases, the gels display superior temperature stability when salt is a component to their compositions. The trend observed is that thermal stability increases from higher (M(III)) to lower oxidation state (M(I)) metal salts (where M is the abbreviation for the metal) and from smaller anions (Cl^−^) to larger anions (Br^−^).

### 4.4. Kinetics with Al(III)

**Figure 3:**
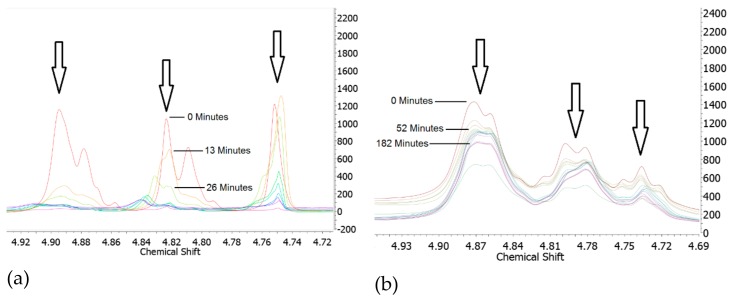
(**a**) Model system without aluminium at 0 °C. (**b**) Model system with aluminium at −25 °C.

NMR studies of model systems of the dynamic aminal junction in the polymer network reveal the effect of reaction rate enhancement in the presence of aluminium. NMR spectra for these studies are presented in Figure 3 and in the supporting information in Figures S2 through S7. Because of the apparent lability of the various intermediates in the condensation pathway, with lower molecular weight amines (such as the butyl amine studied in this model), the temperature under which the experiment was monitored was lowered to −25 and 0 °C.

At −25 °C, the species in the system without aluminium remain static. However, the addition of aluminium to this system at this temperature affords us a transformation of these species. It is not until raising the temperature of the system up to 0 °C when we begin to see transformation of species in the system without aluminium. Figure 3a shows the spectral region from 4.92 ppm to 4.72 ppm. ^1^H NMR peaks in these regions in a similar model system (with polypropylene glycol amine in place of the butylamine used here) have been previously correlated to R–NH–CH_2_–OH functionalities. These transformations occur over approximately 30 min at 0 °C. The depletion of these peaks indicates the conversion of hemiaminal in to aminal (hexahydrotriazine) [11]. Figure 3b shows the spectral region from 4.93 to 4.69 ppm where the R–NH–CH_2_–OH functionalities of the aluminium catalysed system deplete over 3 h at −25 °C. The depletion of these peaks also indicates the conversion of hemiaminal in to aminal (hexahydrotriazine), but at a lower temperature than the sample without aluminium. In both cases, the temperature was subsequently raised to 0 °C. At this temperature, the aluminium catalysed system displayed relatively little change, whereas the system without aluminium began to evolve new species corresponding to intermediates along the condensation pathway including the final HT product. 

### 4.5. Computational Analysis

**Scheme 5:**
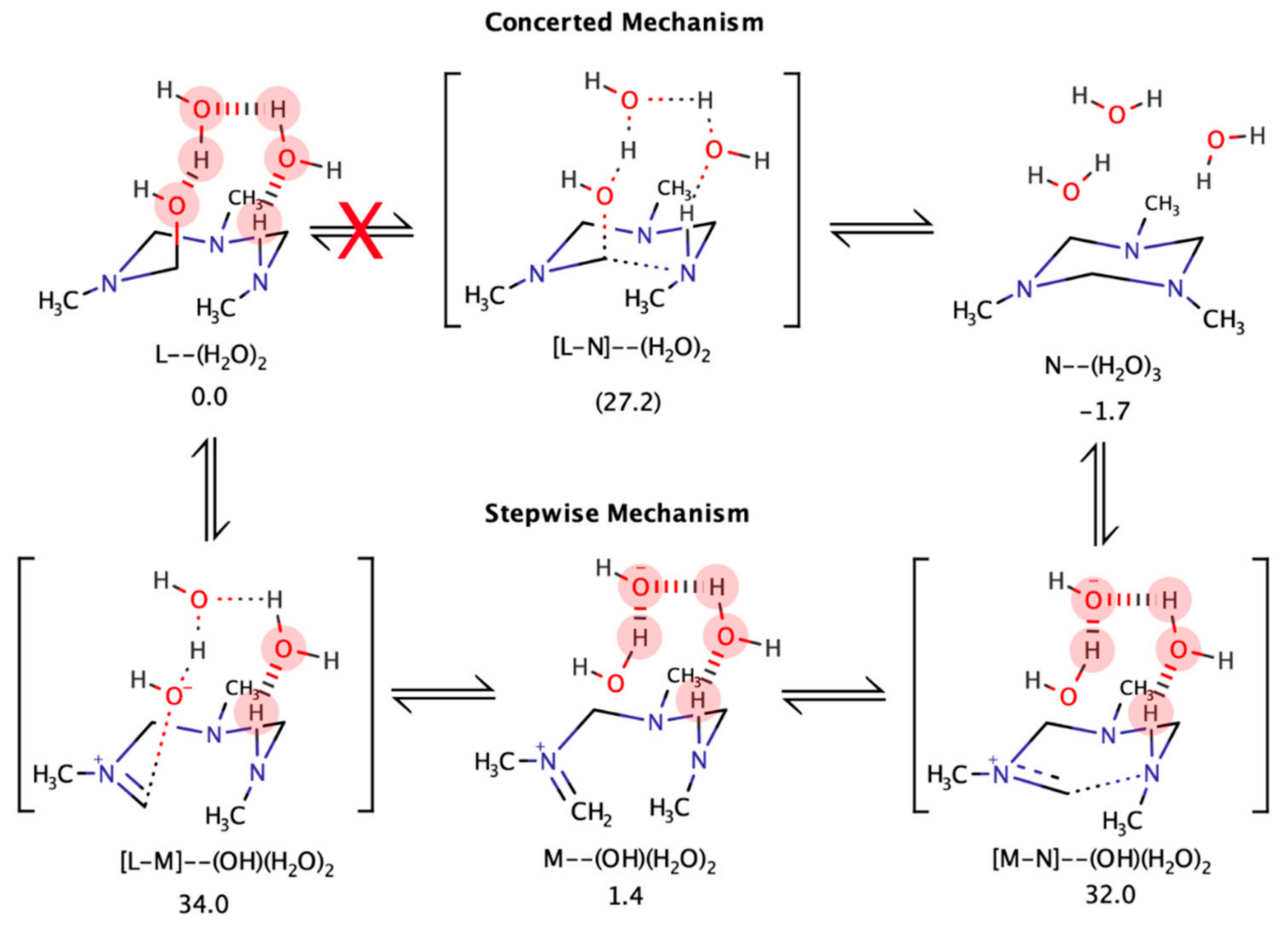
Concerted mechanism of formation of the triazane ring compared to our stepwise mechanism.

The original mechanism of hemi-aminal gel formation proposed by Jones and co-workers [15] is shown in Scheme 5 with the reaction of methylamine and formaldehyde (R = CH_3_). In the Jones mechanism, the rate-determining step (RDS) is described as a concerted loss of water in conjunction with ring formation. The L intermediate (same as in Scheme 2) is considered in the presence of two explicit water molecules that form a hydrogen bonding network linking the hydroxy group at one end and the secondary amine at the other in a pre-activated conformation readied for cyclization. This network facilitates the loss of water by stabilizing the forming charge of the displacing hydroxide anion during cyclization to form water. Two hydrogen bonds support this anion, while simultaneously removing the proton on the secondary amine that is involved in the ring formation to give the final product, N. This transformation proceeds through a concerted transition-state (TS), [L-N].

**Figure 4:**
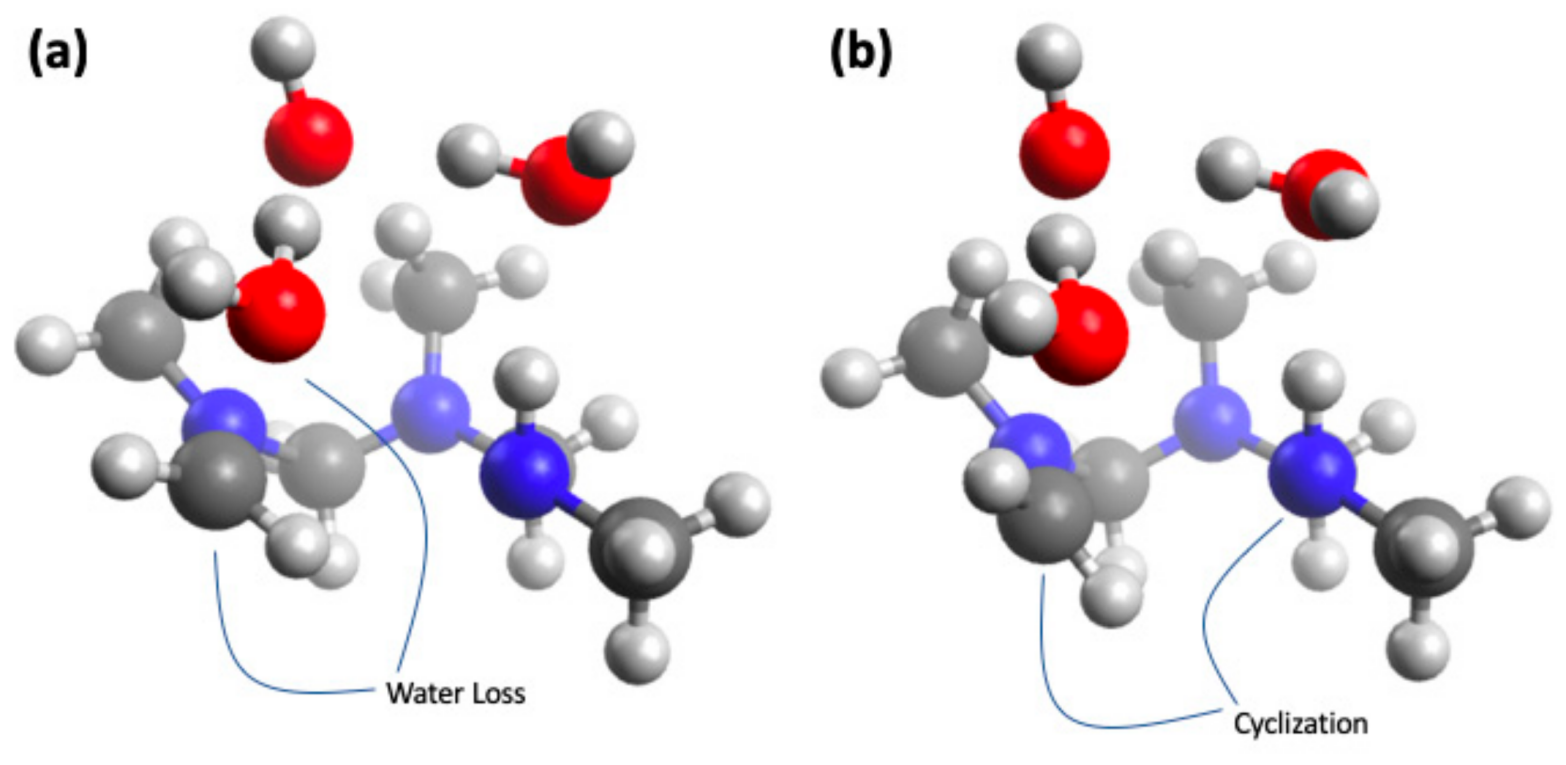
Key structure for the metal-free mechanism. (**a**) [L-M] rate determining step and (**b**) second cyclization transition state [M-N].

Starting from the optimized structure published by Jones (B3LYP), we re-optimized at the B3LYP/6-31+g(d) structural level. We note that Jones’ approach and ours differs only in the use of COSMO in this work compared with the alternative solvent model PCM. We have found that while the proposed transition-state can be localized, our interpretation of its nature is quite different. In examining the imaginary frequency, we find that only small displacements corresponding to ring formation are present, and thus the transition-state proposed in Scheme 4 [L-N]—(H_2_O)_2_ is not a clean cyclization, as it expresses severe asynchronicity. The transition-state we optimized is dominated by the water loss vibrations with small coupled movements representing ring formation. We also performed a full optimization and frequency analysis including COSMO at each interaction to verify our results, yielding no significant difference in outcome. Thus, we propose the transition-state structure above, [L-M]—(H_2_O)_2_, giving intermediate M—(OH)(H_2_O)_2_. M—(OH)(H_2_O)_2_ is an iminium intermediate stabilized by an internal hydrogen bond, which is thus only slightly endergonic (1.4 kcal/mol) and is obtained through a barrier higher compared with Jones (27.2) at 34.0 kcal/mol. Barriers are determined using the M06-2x/6-31+g(d) single-point with COSMO solvation in water. Furthermore, the subsequent ring closure transition-state was localized [M-N]—(OH)(H_2_O)_2_ (small imaginary frequency ca. −80 cm^−1^) having a slightly lower barrier relative to the M—(OH)(H_2_O)_2_ at 32.0 kcal/mol. Thus, the RDS for the, now stepwise, reaction remains the water loss step, but the ring closure step has been decoupled. This is important for understanding the Al catalysed process. Key structures are presented in Figure 4.

**Scheme 6:**
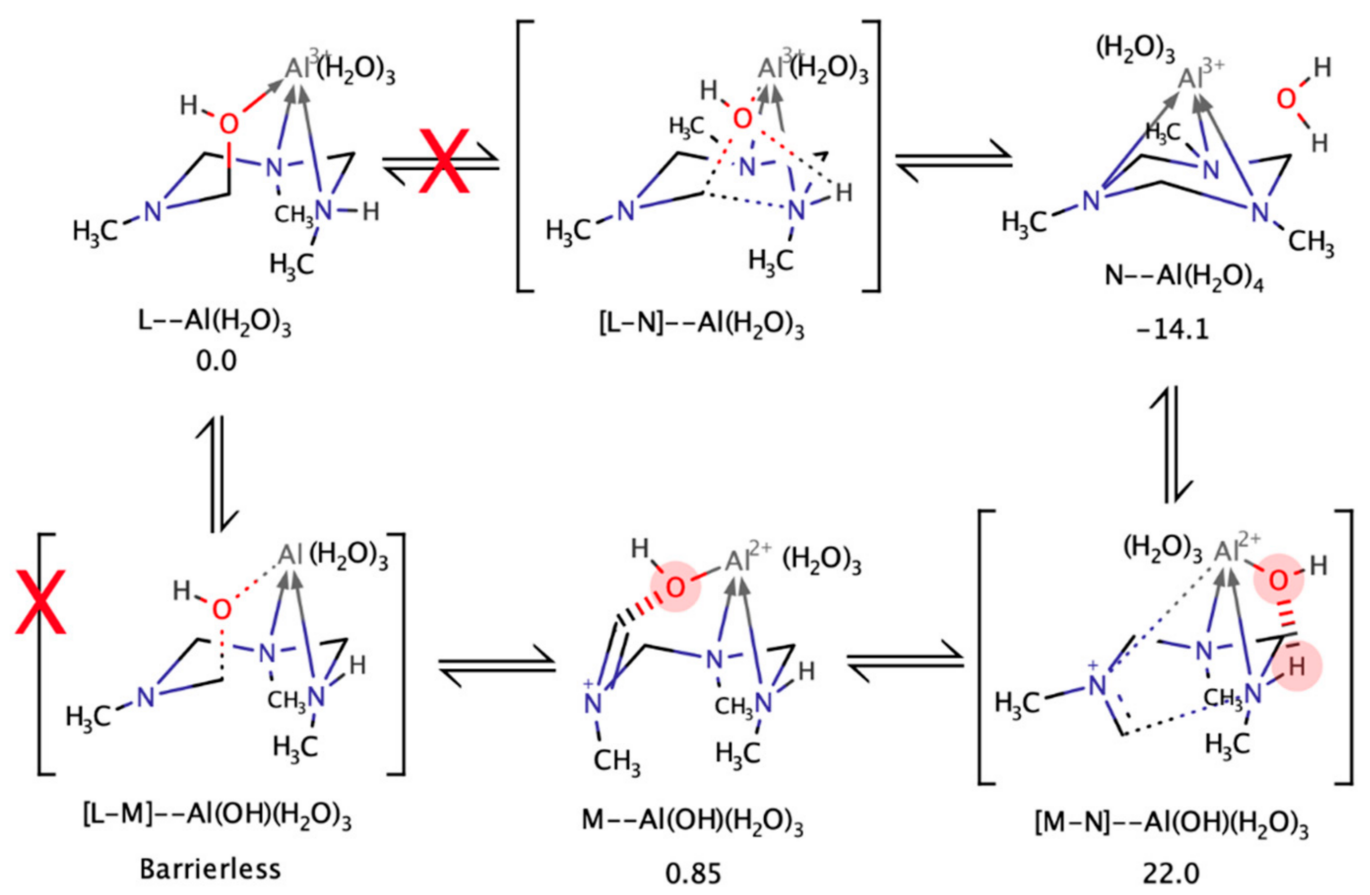
Ring closure mechanism in the presence of aluminium. The red cross on the bracket for [L-M]—Al(OH)(H_2_O)_3_ indicates that the structure is not a stationary point.

In the presence of aluminium, the mechanism alters significantly. The full mechanism is shown in Scheme 6.

**Figure 5:**
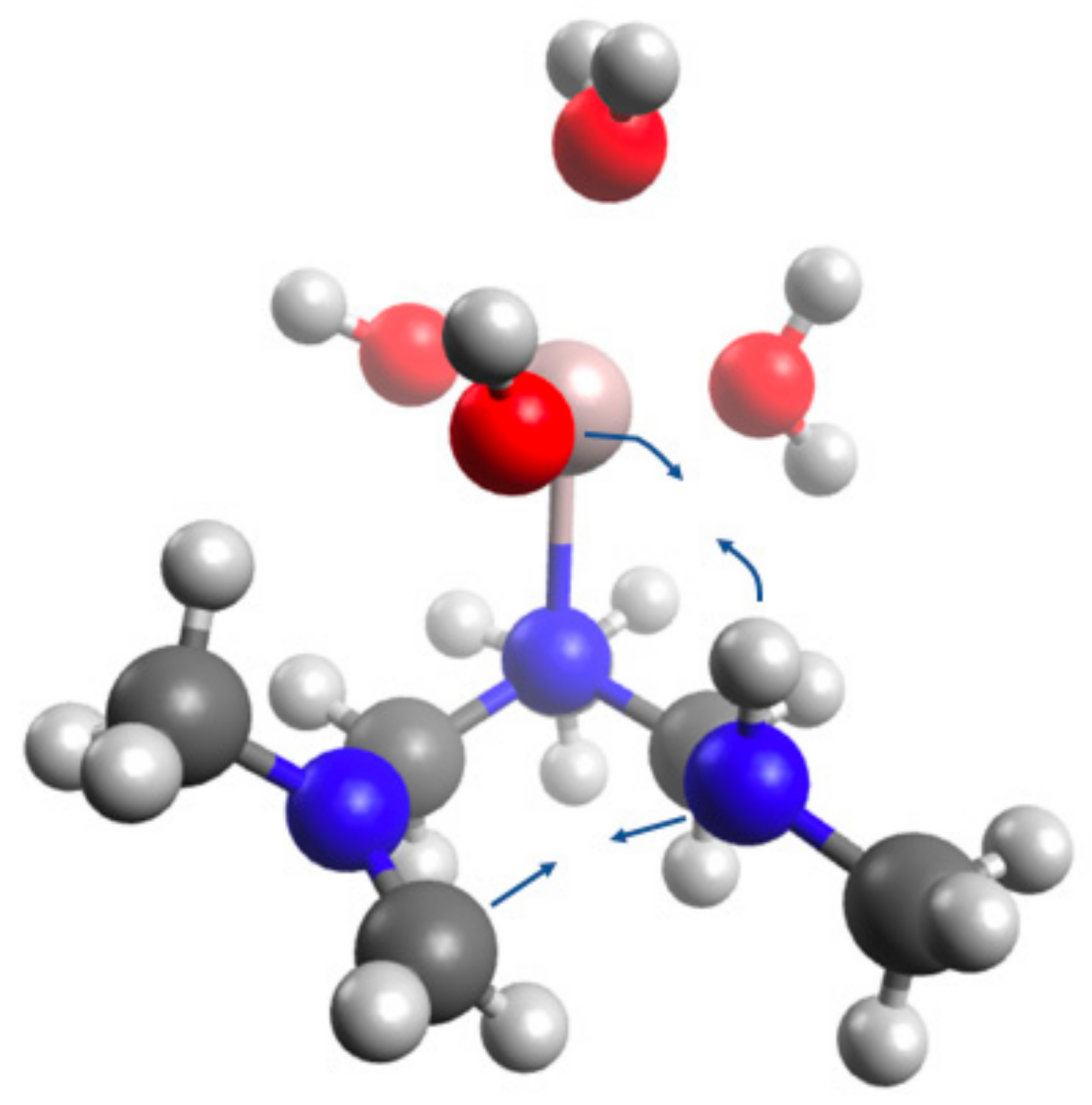
Key rate determining transition-state of aluminium catalysed process with arrows indicated displacement vectors of atoms. Cyclization is accompanied with deprotonation by hydroxide at secondary amine.

In this mechanism, the water molecules are effectively replaced by an aluminium atom coordinated to three water molecules. L—Al(H_2_O)_3_ is a tridentate complex involving two coordinative bonds from the nitrogen atoms and one coordinative bond from the hydroxyl group. The distal nature of the water molecules hinders their direct involvement in the transition-state as part of a hydrogen bonding network (as above). It was not possible to locate the transition-state [L-N]—Al(H_2_O)_3_ leading directly, in a concerted fashion, to the final product in analogy to the Jones mechanism. In the absence of such a TS, we looked extensively for the TS [L-M]—Al(H_2_O)_3_ that would represent loss of hydroxyl with the formation of an aluminium hydroxide, this hydroxyl migration process appears to be barrierless at this level of theory. Thus, we believe M—Al(OH)(H_2_O)_3_ and L—Al(H_2_O)_3_ to be in equilibrium and in roughly equal amounts. Interestingly, the product expresses also a strong hydrogen bond between the iminium methylene group and the aluminium hydroxide group (see Figure 5).

When aluminium is present, the RDS now becomes the ring closure step [M-N]—Al(OH)(H_2_O)_3_ with a barrier of 22.0 kcal/mol. Thus, aluminium acts as a catalyst with respect to the metal-free process. It should also be noted that the overall process is considerably more exothermic at −14.1 kcal/mol compared with −1.7 kcal/mol for the metal-free.

## 5. Conclusions

The catalytic formation of PHT is possible with aluminium chloride and other metals. We believe this catalytic activity to be the result of the coordination of aminal functional groups to metal centers (empty 3s orbitals in the case of aluminium). The presence of aluminium reduces the energetic barrier of the transition from product L to N by 12.4 kcal/mol. Other metal systems such as iron (II) or (III) also show this propensity to catalyze ring closure to HT and display similar non-equilibrium dynamics prior to reaching the thermodynamic product. Catalysis provides one way to achieve the gel formation without heating the materials to temperatures in excess of 150 °C.

This chemical system allows the control of a dynamic non-equilibrium system and operationally instructed sol-gel and gel-sol transformations through dynamic covalent chemistry and the accessibility of constitutional dynamics rendered through the reactivity and coordination of hemiaminal and aminal functionalities. The temperature tolerance of these gels makes them competitive with commercially available solutions in oil well construction materials. Furthermore, there is a rich chemistry that is available in this system by tailoring the R-spacer groups between the reactive amine starting materials. We have identified this aspect of the chemistry with a comparison of PPG and PEG groups, however, many further possibilities remain in exploiting these aspects of this system.

## Figures and Tables

**Table 1:** Gel and liquid formulations with *N*-methyl pyrrolidone (NMP).

65—5 Gel Formulation			
Material	Weight	Molar Ratio	Moles
N-methyl-2-pyrrolidone (NMP)	41.2 g	64.9	0.416
Paraformaldehyde	1.04 g	5.4	0.0346
Jeffamine T- 5000	32.0 g	1	0.0064
Aluminium Chloride Hexahydrate	1.85 g	1.2	0.0077

**Table 2:** Gel and liquid formulations with NMP and Jeffamine ED900.

Gel Formulation with Jeffamine ED900			
Material	Weight	Molar Ratio	Moles
N-methyl-2-pyrrolidone (NMP)	41.2 g	47.8	0.416
Paraformaldehyde	1.04 g	3.9	0.0346
Jeffamine ED900	7.8 g	1	0.0087
Aluminium Chloride Hexahydrate	0.25 g	0.012	0.0001

**Table 3:** Comparison of polyhexahydrotriazine (PHT) gel breakdown.

Breakdown of PHT Gel				
Amine Used in PHT Gel	Phosphine	Phosphine + NMP	Strong Acid + Heat	DI H2O
Polyoxyethlyene Diamine (ED900)	Yes	Yes	Yes	Yes
Polyoxyproplyene Triamine (T5000)	Yes	Yes	Yes	No

**Table 4:** Density of NMP salt solutions.

NMP Salt Solution Table	
Salt Used	Density (g/cc)
Aluminium Chloride Hexahydrate	1.11
Calcium Bromide Hydrate	1.25
Calcium Chloride Dihydrate	1.17
Calcium Chloride Hexahydrate	1.22
Cesium Chloride	1.21
Sodium Bromide	1.05
Zinc Bromide	1.35

**Table 5:** Summary of PHT stability.

High Temperature Thermodynamic Gel Stability					
Salt Catalyst Used	PHT Gel at 70 °C	Stability at 150 °C			
		1 h	24 h	3 Days	7 Days
No Salt	Yes	No	-	-	-
Aluminium Chloride Hexahydrate	Yes	No	-	-	-
Calcium Bromide	Yes	No	-	-	-
Cesium Chloride	Yes	No	-	-	-
Zinc Bromide	Yes	Yes	No	-	-
Calcium Chloride Dihydrate	Yes	Yes	Yes	No	-
Calcium Chloride Hexahydrate	Yes	Yes	Yes	No	-
Sodium Bromide	Yes	Yes	Yes	Yes	Yes

Once a gel system lost stability, no further testing was continued at 150 °C.

## Supplementary Materials

The following are available online at https://www.mdpi.com/2073-4360/11/8/1237/s1, Figure S1. Pictures of the gels depict: (a) the kinetic hemiaminal gel (product V in Scheme 3), (b) the liquid resulting from the addition of aluminium chloride to the kinetic hemiaminal gel (product VII in Scheme 3), (c) the thermodynamically favoured hexahydrotriazine gel after heating the liquid in (b) (product VIII in Scheme 3); and (d) the liquid following breakdown of the gel with tris-carboxylethyl phosphine; Figure S2. PHT system without aluminum; Figure S3a. PHT system without aluminum; Figure S3b. Without aluminum at −30 °C with time resolution; Figure S4. Without aluminum at 0 °C with time resolution; Figure S5. With aluminum with time resolution; Figure S6. With aluminum with time resolution; Figure S7. With aluminum with time resolution.
