# Supplementary material for: Coordination Dynamics and Thermal Stability with Aminal Metallogels and Liquids
Source: Polymers (Basel). 2019 Jul 26;11(8):1237. doi: 10.3390/polym11081237 (PMC6723235; doi:10.3390/polym11081237)
Supplement: Supplementary file 1 [file polymers-11-01237-s001.pdf]

# Coordination Dynamics and Thermal Stability with Aminal Metallogels and Liquids

Peter J. Boul,* Diana K. Rasner, Peter D. Jarowski, and Carl J. Thaemlitz

Aramco Services Company – Aramco Research Center – Houston, 16300 Park Row Blvd. Houston, TX. 77084
* Correspondence: e-mail@e-mail.com

# Contents

Sol-Gel System ........ 3
NMR Model System ........ 4
PHT System Without Aluminum ........ 4
PHT System With Aluminum ........ 7

## Sol-Gel System

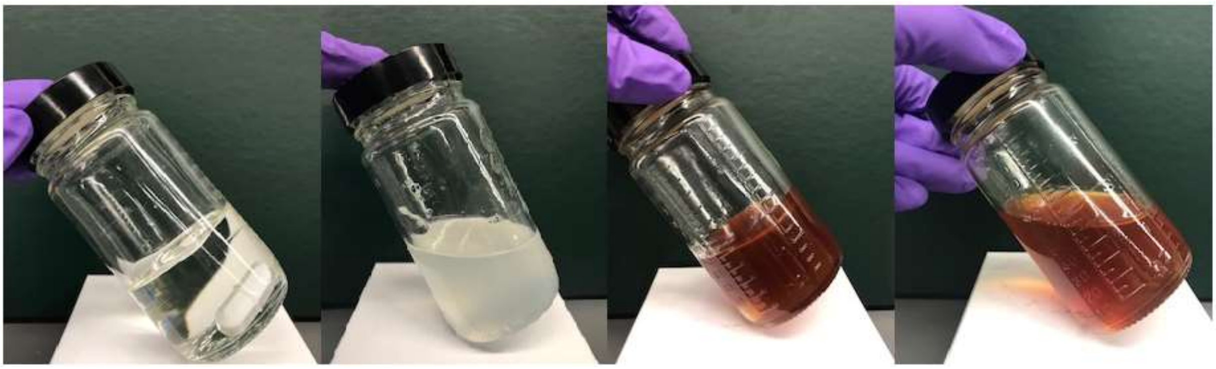

**Figure S-1.** Pictures of the gels depict: (a) the kinetic hemiaminal gel (product V in Scheme 3), (b) the liquid resulting from the addition of aluminium chloride to the kinetic hemiaminal gel (product VII in Scheme 3), (c) the thermodynamically favoured hexahydrotriazine gel after heating the liquid in (b) (product VIII in Scheme 3); and (d) the liquid following breakdown of the gel with tris-carboxylethyl phosphine.

# NMR Model System

## PHT System Without Aluminum

0 Hours : $^1$H NMR (500 MHz, DMF) δ 11.51, 8.03, 7.01, 5.04, 4.91, 4.88, 4.82, 4.80, 4.74, 4.71, 4.63, 4.34, 4.15, 3.80, 3.27, 3.21, 3.04, 2.91, 2.77, 2.73, 2.56, 2.38, 2.32, 2.18, 2.04, 1.67, 1.50, 1.42, 1.41, 1.39, 1.38, 1.36, 1.30, 1.29, 1.27, 1.26, 1.24, 0.87, 0.86, 0.84

1.5 Hours: $^1$H NMR (500 MHz, DMF) δ 11.49, 8.03, 7.01, 7.00, 6.99, 6.84, 4.91, 4.88, 4.86, 4.82, 4.79, 4.78, 4.71, 4.63, 4.34, 4.15, 3.83, 3.79, 3.27, 3.23, 3.21, 3.20, 3.03, 3.03, 2.91, 2.77, 2.73, 2.56, 2.39, 2.38, 2.36, 2.31, 2.18, 2.04, 1.67, 1.52, 1.50, 1.50, 1.49, 1.42, 1.41, 1.39, 1.38, 1.36, 1.30, 1.29, 1.27, 1.26, 0.86.

2.5 Hours: $^1$H NMR (500 MHz, DMF) δ 8.03, 4.89, 4.88, 4.82, 4.81, 4.75, 4.39, 4.36, 4.23, 4.15, 4.09, 4.05, 3.58, 3.53, 3.46, 3.38, 3.23, 3.21, 3.10, 3.01, 2.92, 2.75, 2.69, 2.68, 2.67, 2.65, 2.61, 2.59, 2.57, 2.56, 2.54, 2.52, 2.51, 2.50, 2.39, 2.37, 2.30, 2.17, 2.03, 1.40, 1.39, 1.36, 1.34, 1.33, 1.31, 1.30, 1.29, 0.90, 0.89, 0.88, 0.87, 0.86.

10.5 Hours: $^1$H NMR (500 MHz, DMF) δ 8.03, 6.85, 4.63, 3.53, 3.45, 3.44, 3.10, 3.01, 2.96, 2.92, 2.92, 2.92, 2.79, 2.75, 2.75, 2.74, 2.61, 2.60, 2.58, 2.57, 2.57, 2.55, 2.54, 2.52, 2.51, 2.49, 2.39, 2.38, 2.36, 2.30, 2.18, 2.17, 1.42, 1.42, 1.41, 1.40, 1.39, 1.39, 1.38, 1.38, 1.37, 1.36, 1.34, 1.33, 1.31, 1.30, 1.30, 1.30, 1.28, 1.27, 0.90, 0.89, 0.89, 0.88, 0.88, 0.87, 0.87, 0.86, 0.85.

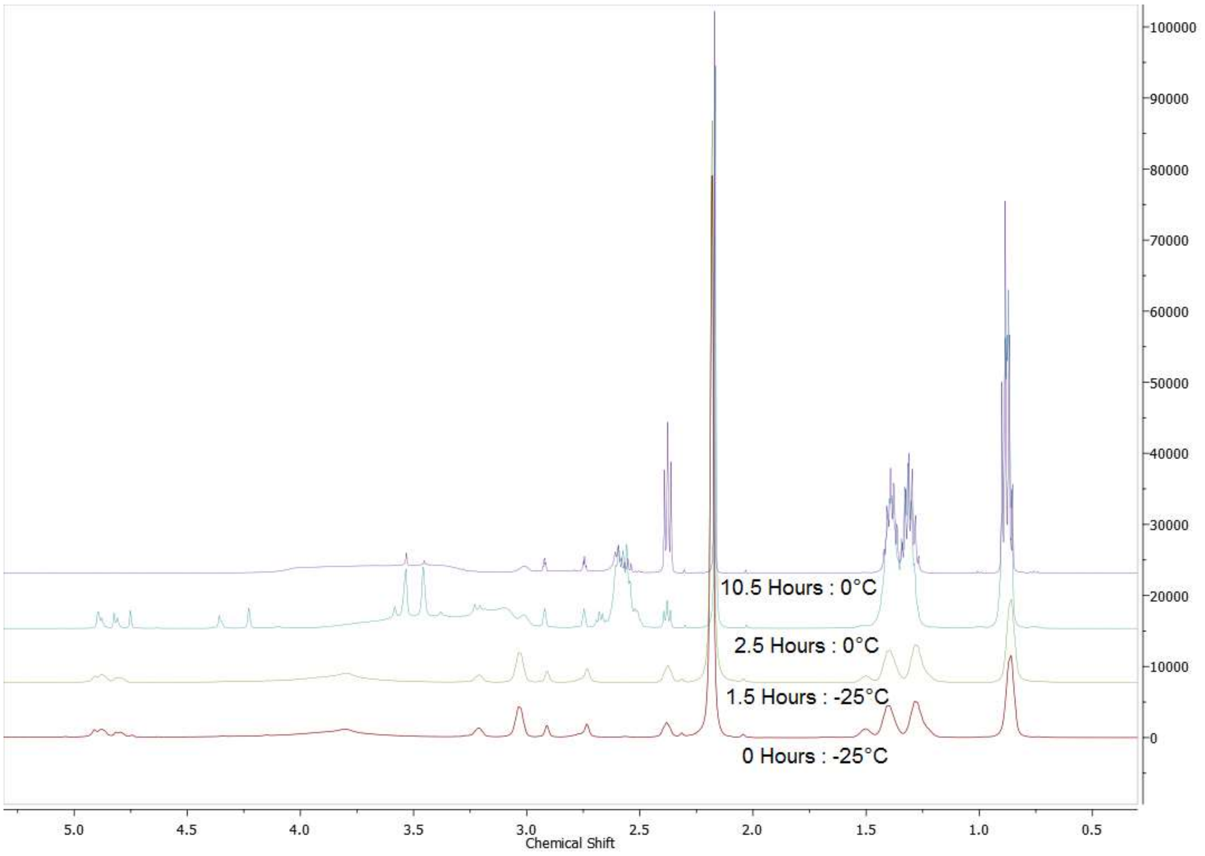


**Figure S-2**. PHT system without aluminum.

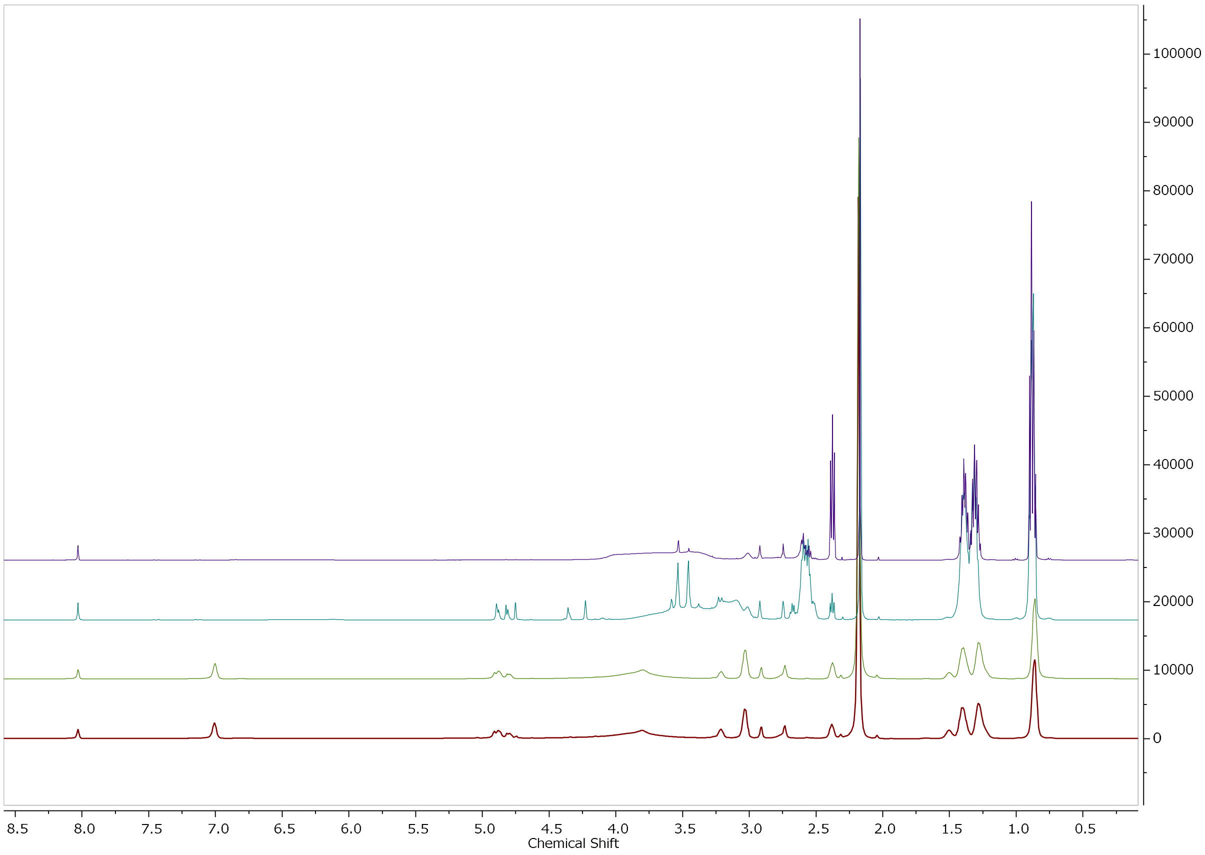


**Figure S-3**. PHT system without aluminum.

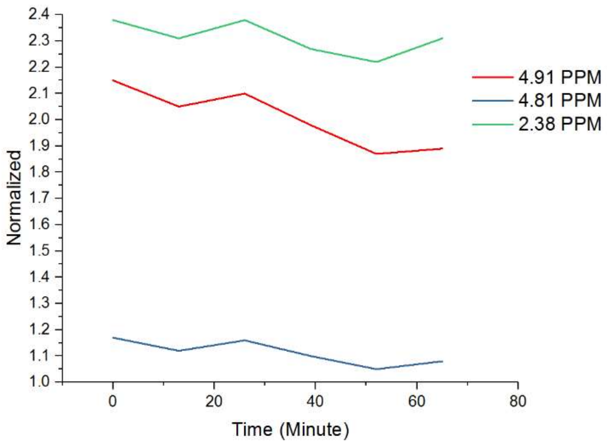


**Figure S-3**. Without aluminum at -30°C with time resolution.

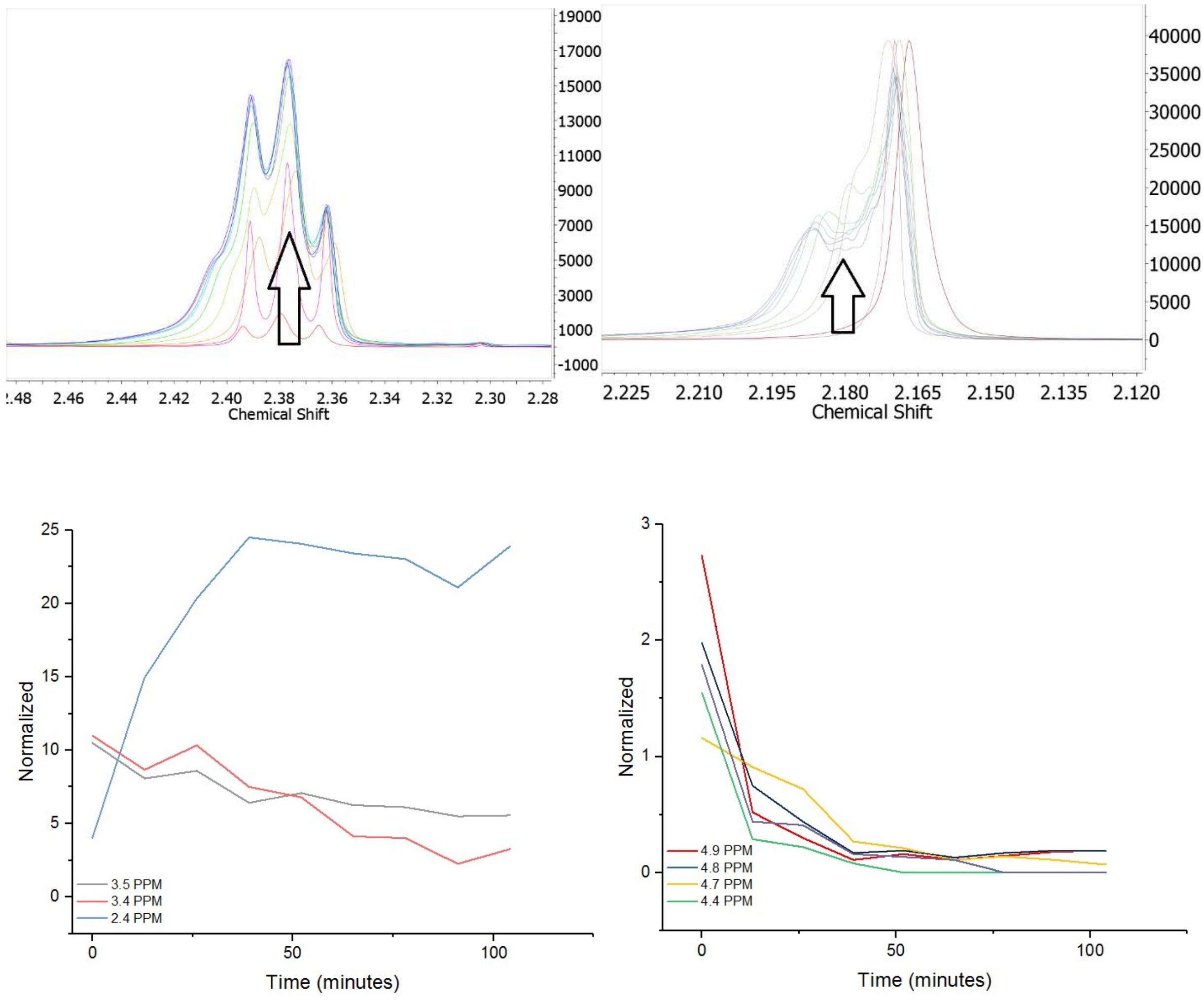


**Figure S-4**. Without aluminum at 0°C with time resolution.

# NMR Model System

## PHT System With Aluminum

0 Hours: $^{1}$H NMR (500 MHz, DMF) δ 9.66, 8.61, 8.11, 8.03, 6.84, 6.72, 6.50, 6.22, 6.21, 6.19, 5.41, 4.88, 4.87, 4.86, 4.83, 4.82, 4.80, 4.78, 4.75, 4.74, 4.72, 4.66, 4.64, 4.51, 4.49, 4.41, 4.36, 4.34, 3.80, 2.97, 2.91, 2.77, 2.73, 2.19, 2.13, 1.71, 1.42, 1.26, 0.86.

6 Hours: $^{1}$H NMR (500 MHz, DMF) δ 8.69, 8.03, 6.99, 6.88, 6.21, 5.78, 4.87, 4.86, 4.80, 4.78, 4.73, 3.84, 3.81, 3.02, 3.01, 2.98, 2.96, 2.95, 2.91, 2.72, 2.56, 2.34, 2.19, 1.70, 1.68, 1.36, 1.25, 0.85.

7 Hours: $^{1}$H NMR (500 MHz, DMF) δ 8.65, 8.03, 6.85, 6.73, 6.06, 5.38, 4.89, 4.87, 4.80, 4.75, 4.33, 3.70, 3.68, 3.32, 3.27, 3.04, 3.03, 2.99, 2.98, 2.96, 2.92, 2.74, 2.58, 2.35, 2.31, 2.17, 1.74, 1.72, 1.71, 1.43, 1.42, 1.40, 1.38, 1.36, 1.31, 1.30, 1.28, 1.26, 0.91, 0.89, 0.87, 0.84

15 Hours: $^{1}$H NMR (500 MHz, DMF) δ 8.67, 8.03, 6.86, 6.76, 6.08, 6.01, 5.88, 4.89, 4.81, 4.80, 3.69, 3.03, 2.98, 2.96, 2.95, 2.92, 2.86, 2.74, 2.66, 2.47, 2.19, 2.17, 2.10, 1.74, 1.72, 1.70, 1.63, 1.41, 1.39, 1.38, 1.31, 1.30, 1.28, 1.25, 0.89, 0.87, 0.80

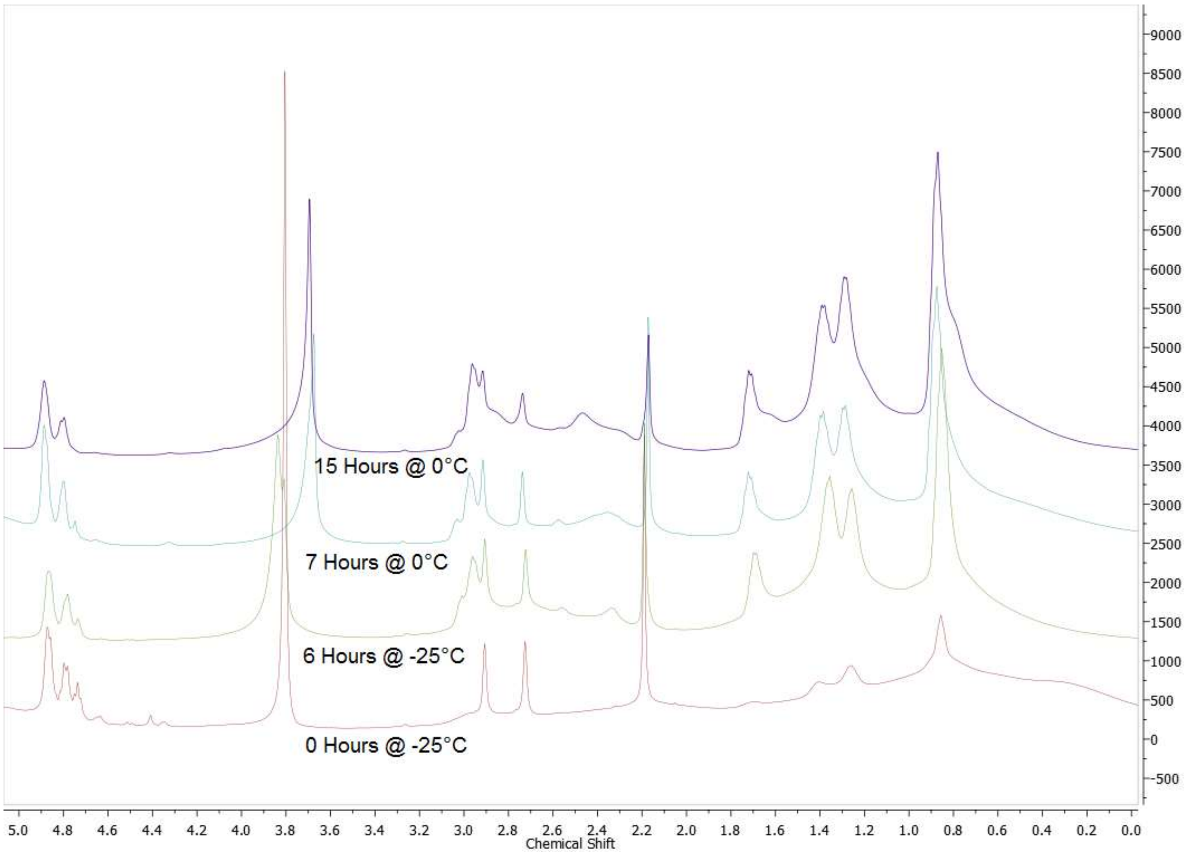


**Figure S-5**. With aluminum with time resolution.

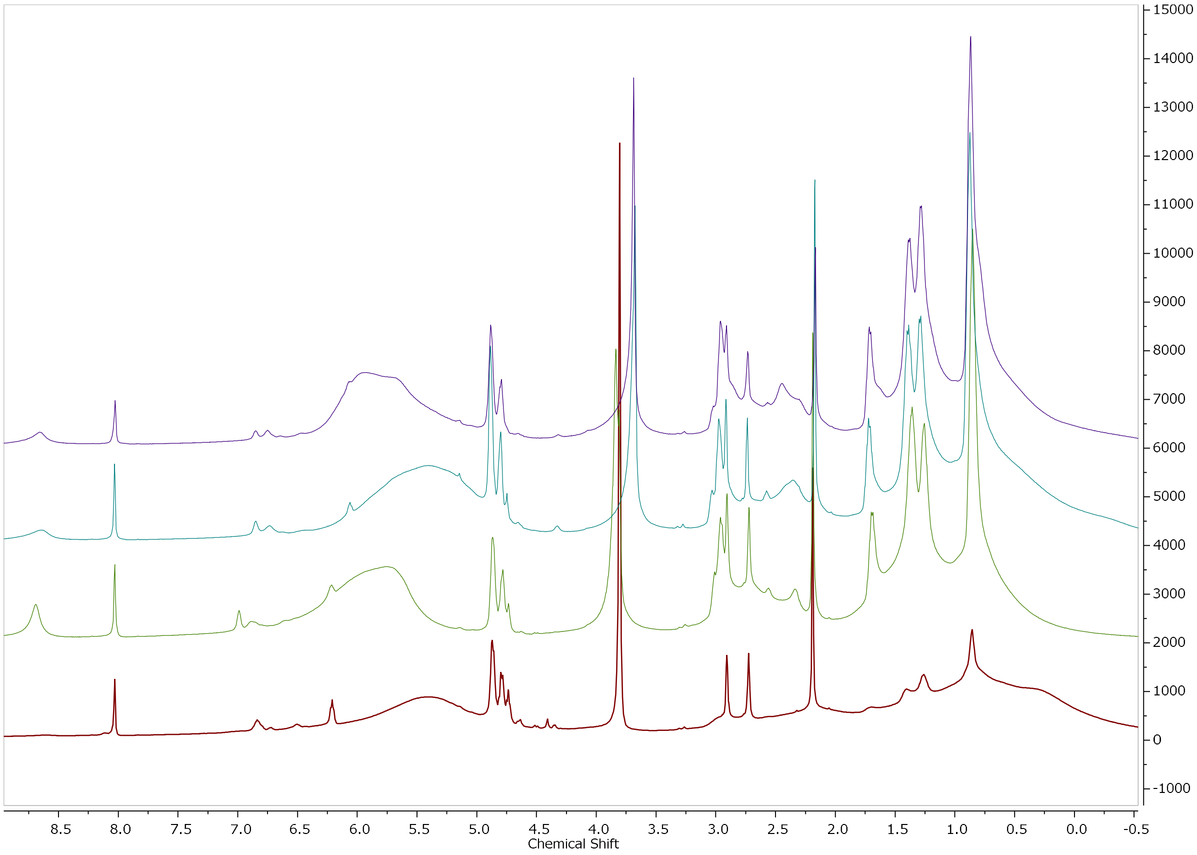


**Figure S-6**. With aluminum with time resolution.

**With Aluminum @ -25°C Overtime**

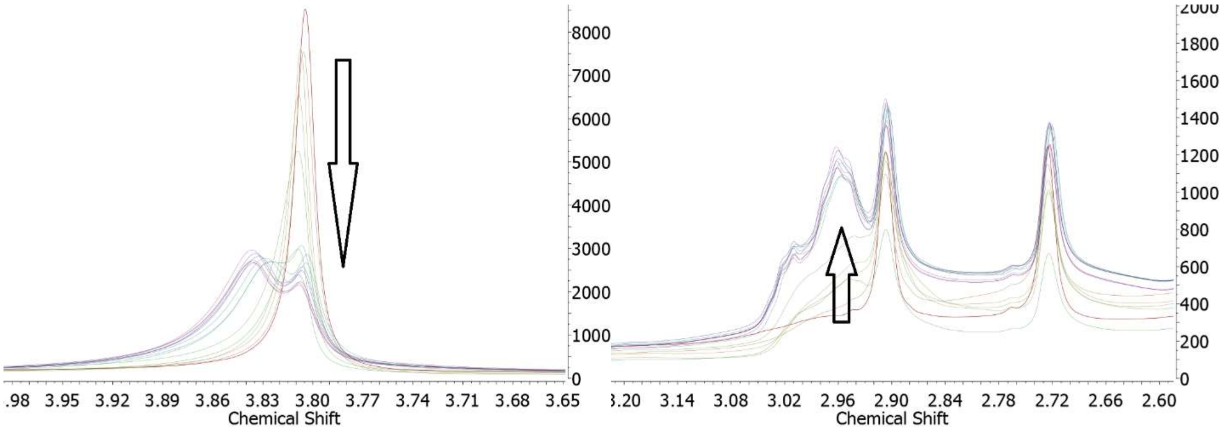

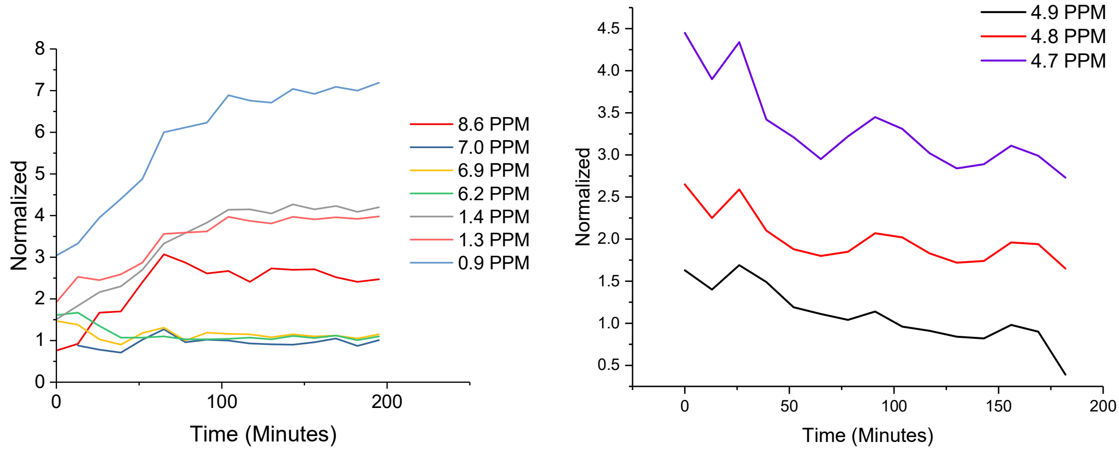


**Figure S-7**. With aluminum with time resolution.
